# Supplementary material for: Decrease in decision noise from adolescence into adulthood mediates an increase in more sophisticated choice behaviors and performance gain
Source: PLoS Biol. 2024 Nov 14;22(11):e3002877. doi: 10.1371/journal.pbio.3002877 (PMC11563475; doi:10.1371/journal.pbio.3002877)
Supplement: S1 Text — File providing additional information on data analysis and results including mixed and computational models as well as correlational analysis and the employed tasks. (PDF) [file pbio.3002877.s001.pdf]

Supplemental Materials - Table of Contents

Data analysis and results 1
Sample Characteristics 1
General task performance 1
Mixed-effects models 2
Mixed-effects model analysis of non-selective Pavlovian biases (DV = PGo) 2
Mixed-effects model analysis of non-selective Pavlovian biases (DV = Pcorrect) 2
Mixed-effects model analysis of selective instrumental learning biases 2
Alternative mixed-effects model capturing instrumental learning biases 2
Control analysis: Gender effects 3
Computational modelling 3
Age effects on computational model parameters 3
Computational modelling: model space: 4
Assessment of model performance using simulations 4
Parameter recovery based on simulated data (M7) 5
Mediation effects of noise on task performance 5
Probabilistic reversal task 5
Sequential (2-step) task 5
Cross-task correlations 6
Correlational analysis of unspecific noise with higher-level cognition 6
Go/NoGo task 6
Reversal task: 6
2-step task: 6
References 8

Data analysis and results

Sample Characteristics

For a better characterization of or sample, we assessed both the age and gender distribution of our participants. Results for this are visualized in Figure S1.

General task performance

Participants' overall probability of executing the correct response for a given cue was 66% (P(correct) mean [SD] = 66.24 [0.18], median [SD] = 69.69 [0.18]). When further assessing task performance based on bias congruency, it became evident that P(correct) for bias-congruent cues (mean [SD] = 70.50 [0.19]) was higher than for bias-incongruent cues (mean [SD] = 65.63 [0.19]). Such finding was to be expected, given that participants have to overcome and actively suppress their biased action tendency on bias-incongruent trials, making those trials more challenging than bias-congruent ones.

Mixed-effects models

Mixed-effects model analysis of non-selective Pavlovian biases (DV = PGo)

The mixed-effects model assessing age effects of Pavlovian biases showed well-known task effects such as successful task learning and the impact of Pavlovian biases on choices, i.e. more Go responses for cues yielding rewarding outcomes vs. more NoGo responses for punishment cues (see table S1 for complete model statistics).

As can be derived from the model statistics in table S1, we see a trend for the three-way interaction between required action x valence x age (Figure S3A). As the interaction term for required action x valence usually indicates a difference in the strength of the approach relative to the avoidance component of Pavlovian bias, we further explored how this trend-wise effect looked like when assessed for age-dependent changes. When plotting the averaged raw data capturing those two different components, namely PGo (G2W -G2A) for the Approach Bias and PGo (NG2W -NG2A) for the Avoid Bias, it can be seen that the Approach Bias is subject to age-dependent changes (Figure S1C/D).

Mixed-effects model analysis of non-selective Pavlovian biases (DV = Pcorrect)

An alternative way to investigate age effects on Pavlovian biases is the assessment of accurate performance (DV = Pcorrect) using mixed-effects models. Here, the effect of interest, namely the developmental influence, is represented by the three-way interaction valence x required action x age. A significant main effect of age indicates that the older participants get the more Go responses they show ($p = 0.04$). Also, the interplay of valence and required action, i.e. whether a cue's action requirement is aligned with the Pavlovian bias, such as Go cues for a reward cue, or not, impacts the level of accuracy as well. However, the three-way interaction including age falls short of becoming significant ($p = 0.19$), despite evidence from the theoretically equivalent model using PGo as DV. This is presumably due to a lack of power to detect this more complex three-way interaction effect (see table S2 for model statistics).

Mixed-effects model analysis of selective instrumental learning biases

To complete reporting of the results for the second mixed-effects model assessing age-dependent changes for selective instrumental learning biases from the main manuscript, see table S3 for full model statistics.

Alternative mixed-effects model capturing instrumental learning biases

We also used a different model equation to capture the instrumental learning bias, which relied more on factors known from the basic model assessing the presence of the Pavlovian bias in the data. Namely, we set up a model testing whether the probability of repeating a previous response, P(repeat), changed depending on three within-subject factors, namely the response shown on the previous trial (Go vs. NoGo), the cue valence (Win vs. Avoid Punishment) and

outcome valence (positive: reward for reward cues; neutral feedback for avoidance cues vs. negative: punishment for avoidance cues; neutral feedback for reward cues). Importantly, in this model, the presence of a learning bias would be indicated by a significant 2-way interaction action shown x outcome valence:

$$P(\text{repeat}) \sim \text{action.shown}_{t-1} * \text{outcome.valence}_{t-1} * \text{cue.valence}_{t-1} + (\text{action.shown}_{t-1} * \text{outcome.valence}_{t-1} * \text{cue.valence}_{t-1} + 1|\text{Subject})$$

Control analysis: Gender effects

To verify potentially relevant effects of gender on the effects of interest, namely the impact of age on Pavlovian and instrumental learning biases, we assessed two additional models as follows:

Pavlovian Bias:

$$\text{DVgo} \sim \text{valence*action*(age\_z+gender)} + (1+\text{valence*action}|\text{ID})$$

Instrumental learning bias:

$$P(\text{repeat}) \sim \text{action taken}_{t-1} * \text{outcome valence}_{t-1} * \text{outcome salience}_{t-1} *(\text{age\_z} + \text{gender}) + (\text{action taken}_{t-1} * \text{outcome valence}_{t-1} * \text{outcome salience}_{t-1} + 1|\text{Subject})$$

In each case, the pattern and direction of the main and interaction effects did not change and none of the effects significantly interacted with gender (Table S4 and S5).

Computational modelling

Age effects on computational model parameters

For completeness, figure S4 depicts the correlational scatterplots for all model parameters and age, independent of the statistical significance of the correlational coefficients.

As an additional sanity check for the validity of our winning model, we ran a regression analysis examining whether outcome valence, i.e. whether participants received a negative or positive feedback within the respective reward vs. punishment context (e.g. neutral feedback in a reward trial = negative feedback vs. in a punishment trial = positive feedback) on a specific trial, age and their interaction significantly impacted feedback sensitivity as extracted from our computational model. For this, we employed the following equation: feedback sensitivity ~ outcome valence * age. Here we found a significant main effect of outcome valence (ß = 1.429, SE = 0.39, t = 3.61, $p < 0.001$), a trend for a main effect of age (ß = 0.031, SE = 0.02, t = 1.90, p = 0.059) and a significant interaction term between outcome and age (ß = 0.036, SE = 0.02, t= 2.20, p = 0.03). These results further reinforced our confidence in our modelling findings, as it clearly showed that outcome valence had a distinct impact on feedback sensitivity, i.e.

explained different aspects of its variance, while also demonstrating that its interaction with age explained additional variance above and beyond the individual effects.

As for parameter transformations, some of the feedback sensitivity parameters, namely, ρ, ρ_{Win} *and* ρ_{avoid}, as well as the learning rate parameter κ were constrained to positive values through an exponential transform, while both outcome-dependent feedback sensitivity parameters, ρ_{+FB} or ρ_{-FB}, from model M7 remained untransformed. The learning rate ε was constrained between 0 and 1 (using an sigmoid transform). The other parameters including those capturing the go bias and Pavlovian bias remained untransformed.

Computational modelling: model space:

We assessed seven increasingly complex models, to determine whether increasing complexity, i.e. including additional hypothesized processes such as specific feedback sensitivity parameters for cue or outcome valence would improve model fit. We used a selection of models (M1-M6) shown to capture motivational biases in this task in prior work [1–3] and a new model (M7). Models M6 and M7 were of particular interest to our research question regarding decision noise. While model M6 included two parameters capturing cue-based feedback sensitivity ($\rho_{\text{Win Cue}}$ and $\rho_{\text{Avoid Cue}}$), M7 comprised two inherently different feedback sensitivity parameters that were feedback-based (ρ_{+FB} and ρ_{-FB}). In short, within an RL framework, feedback sensitivity (ρ) formalizes the degree of choice stochasticity or, put more simplistically, decision noise, by computing the maximum disparity between expected values. A smaller feedback sensitivity parameter translates into expected values being less apart from each other and thus more random behaviour. Meanwhile, larger feedback sensitivity means a greater distance between expected values, which in turn translates into more deterministic choices. Importantly, decision noise might be distinctively impacted by the receipt of different feedback properties, such as receiving positive or negative outcomes after specific choices [4].

Assessment of model performance using simulations

In order to assess how well the models included in our model space were able to capture basic behavioral performance in the Go/NoGo task, we simulated n = 100 synthetic datasets based on the established optimal parameter estimates for the more complex models M3-M5 that had already been established in previous publications [1,3,5]. This also served a second purpose, namely, to select for which RL model we would pursue additional parameterization of feedback sensitivity parameters for outcome valence. Based on visual inspection of the observed vs. the simulated data (compare Figure S5), it became evident, that especially the models containing a parameter to capture the instrumental learning bias, performed particularly bad, while models containing parameters to capture both Pavlovian and instrumental learning biases performed equally well to models only comprising a Pavlovian bias parameter. Hence, we chose to pursue

a more parsimonious approach for extending our model space rather than just extending it with all possible models including the new parameters for feedback sensitivity for positive and negative outcomes. This meant only adding the respective new feedback sensitivity parameters to a model including the Pavlovian bias parameter.

Parameter recovery based on simulated data (M7)

Results of the parameter recovery for the winning computational model M7 are depicted in Figure S6.

Mediation effects of noise on task performance

As already outlined in the main manuscript, the computational parameter capturing feedback sensitivity for positive outcomes exerted a mediating effect on the association between age and overall performance accuracy, also termed P(correct), in the Go/NoGo task. This is also displayed in Figure S7.

Probabilistic reversal task

The probabilistic reversal learning task assessed the distinct impact of motivational context on cognitive flexibility by including two distinct task blocks using reward or punishment outcomes. In the reward block, participants were presented with reward and neutral feedback throughout the learning process, while neutral and punishment feedback was presented in the punishment block. Across blocks, participants had to choose between two cards, each associated with a different likelihood of receiving the optimal outcome (reward block: winning 10 cents vs. not winning; loss block: loss of 10 cents or not losing) (80%-20% and 20%-80%, respectively). Based on trial and error, participants had to figure out which cards yielded the better outcome overall. Following an acquisition phase, reward contingencies reverse 5 times, thus requiring participants to continuously update their expectations of the cards` turnover, as the previously best card becomes the less optimal one (see [4].

Sequential (2-step) task

The 2-step task is a well-established probabilistic reinforcement learning task, in which individuals first need to decide between two images in the first stage to get to a second state. After having made this choice, both images lead to one of two second stages following a fixed but probabilistic schedule. One transition occurs more frequently than the other (common vs. rare) and transitions were fixed at 70% vs. 30%. While one first stage image led more frequently to one of the second stages, this was reversed for the other image. In the second stage, participants were again required to make a choice between 2 images, which consequently led to a reward. Importantly, the probability with which participants received a reward was not fixed but followed a slowly, but continuously changing probability schedule (random walk). Thus, in order to maximize reward receipt on this task, participants needed to

constantly track which second state image would yield rewards most frequently at a given moment, while also adjusting their choice behaviour on the first and second stage according to the changing probabilities. Finally, in this task version, there was also a second block that employed punishment instead of rewards, so that participants needed to minimize their losses.

Cross-task correlations

As already outlined in the main manuscript, we assessed cross-task correlations between feedback sensitivity (noise) parameters for positive and negative outcomes from the motivational Go/NoGo task with noise parameters derived from the computational modelling of the reversal task data. Here, we found none of the feedback sensitivity parameters for negative outcomes to be significantly correlated with the reversal task noise parameters, which is also depicted in Figure S8.

Correlational analysis of unspecific noise with higher-level cognition

Go/NoGo task. To determine the extent to which specific task performance on the Go/NoGo task is impacted by unspecific factors such as decision noise, we correlated both decision noise parameters from our winning model M7 (ρ_{+FB} and ρ_{-FB}) with task performance overall (P(correct). Here, more feedback sensitivity for positive outcomes was linked to better task performance [r_s (90) = 0.78, $p < 0.001$], while the opposite pattern was shown for feedback sensitivity for negative outcomes and performance. Here, more feedback sensitivity for negative outcomes was associated with decreased task performance [r_s (90) = - 0.57, $p < 0.001$].

Reversal task: Examining the association between positive and negative feedback sensitivities from the Go/NoGo task and parameters from the reversal task showed that higher positive feedback sensitivity was associated with both increased pre-post reversal accuracy [r_s (87) = 0.44, $p < 0.001$] and less switching behaviour after negative outcomes in the reversal learning task [r_s (87) = -0.44, $p < 0.001$]. Higher feedback sensitivity after negative outcomes was significantly linked to lower pre-post reversal performance [r_s (87) = - 0.27, $p = 0.01$], while the association with switching behaviour after negative outcomes fell short of becoming significant after multiple comparison correction [r_s (87) = 0.24, $p = 0.021$].

2-step task: We then assessed the link between feedback sensitivity for positive and negative outcomes (here less feedback sensitivity represents increased decision noise) with model-based (MB) control. MB control is usually considered a higher-level cognitive function and is described as a mental computation of a ‘world model’ tracking the outcomes and thus values of our actions as well as simulating hypothetical ones for all given states [6]. Thus, as a corollary, we had hypothesized that higher decision noise, for positive and negative outcomes, would be linked to decreased MB control, as it might impede the formation of such cognitive

model or map. As expected, we found evidence that higher feedback sensitivity for positive outcomes was significantly linked to more model-based control [r_s (90) = 0.54, $p < 0.001$] and survived multiple comparison correction (p -value < .025 (0.05/2) considered significant). Feedback sensitivity for negative outcomes was not significantly correlated to MB control [r_s (90) = 0.54, $p = 0.089$].

References

1. **Algermissen J, Swart JC, Scheeringa R, Cools R, den Ouden HEM. Prefrontal signals precede striatal signals for biased credit assignment in motivational learning biases. Nat Commun. 2024;15: 19. doi:10.1038/s41467-023-44632-x**

2. **Scholz V, Hook RW, Kandroodi MR, Algermissen J, Ioannidis K, Christmas D, et al. Cortical dopamine reduces the impact of motivational biases governing automated behaviour. Neuropsychopharmacology. 2022;47. doi:10.1038/s41386-022-01291-8**

3. **Swart JC, Froböse MI, Cook JL, Geurts DEM, Frank MJ, Cools R, et al. Catecholaminergic challenge uncovers distinct Pavlovian and instrumental mechanisms of motivated (in)action. eLife. 2017;6: 1–36. doi:10.7554/eLife.22169**

4. **Waltmann M, Herzog N, Reiter AMF, Villringer A, Horstmann A, Deserno L. Diminished reinforcement sensitivity in adolescence is associated with enhanced response switching and reduced coding of choice probability in the medial frontal pole. Dev Cogn Neurosci. 2023;60. doi:10.1016/j.dcn.2023.101226**

5. **Swart JC, Frank MJ, Määttä JI, Jensen O, Cools R, den Ouden HEM. Frontal network dynamics reflect neurocomputational mechanisms for reducing maladaptive biases in motivated action. PLoS Biol. 2018;16: e2005979. doi:10.1371/journal.pbio.2005979**

6. **Drummond N, Niv Y. Model-based decision making and model-free learning. Curr Biol. 2020;30: R860–R865. doi:10.1016/j.cub.2020.06.051**
