# Supplementary material for: Decrease in decision noise from adolescence into adulthood mediates an increase in more sophisticated choice behaviors and performance gain
Source: PLoS Biol. 2024 Nov 14;22(11):e3002877. doi: 10.1371/journal.pbio.3002877 (PMC11563475; doi:10.1371/journal.pbio.3002877)
Supplement: S1 Table — Table displaying the ß estimates, the standard error (SE) as well as statistics for the main and interaction effects from the mixed-effects model computed to assess the impact of age on Pavlovian biases as well as general learning of the task. Here, the dependent variable was the probability of making a go response P(Go). Data and code to compute the statistics presented in this table is available at https://osf.io/mcx36/. (PDF) [file pbio.3002877.s002.pdf]

	ß estimates	SE	χ^2	p-value
**Main effects**				
valence	0.363	0.05	59.5	<.001 ***
required action	1.423	0.09	238.8	<.001 ***
age	-0.01	0.07	0.03	0.9
**Interaction effects**				
required action x valence	0.161	0.04	13.6	<.001 ***
valence x age	0.107	0.05	5.3	0.02*
required action x age	0.210	0.09	5.0	0.03*
valence x required action x age	0.082	0.04	3.6	0.06^{+}
