# Supplementary material for: Decrease in decision noise from adolescence into adulthood mediates an increase in more sophisticated choice behaviors and performance gain
Source: PLoS Biol. 2024 Nov 14;22(11):e3002877. doi: 10.1371/journal.pbio.3002877 (PMC11563475; doi:10.1371/journal.pbio.3002877)
Supplement: S2 Table — Table displaying the ß estimates, the standard error (SE) as well as statistics from the mixed-effects model computed to assess the impact of age on Pavlovian biases as well as general learning of the task. Here, the dependent variable was the probability of making a correct response P(Correct). Data and code to compute the statistics presented in this table is available at https://osf.io/mcx36/. (PDF) [file pbio.3002877.s003.pdf]

	ß estimates	SE	χ^2	p-value
**Main effects**				
valence	0.037	0.04	0.8	.4
required action	-0.085	0.06	2.3	.1
age	0.205	0.10	4.3	0.04*
**Interaction effects**				
required action x valence	0.238	0.04	33.8	<.001 ***
valence x age	0.029	0.04	0.5	0.5
required action x age	-0.013	0.06	0.05	0.8
valence x required action x age	0.054	0.04	1.7	0.19
