# Supplementary material for: Decrease in decision noise from adolescence into adulthood mediates an increase in more sophisticated choice behaviors and performance gain
Source: PLoS Biol. 2024 Nov 14;22(11):e3002877. doi: 10.1371/journal.pbio.3002877 (PMC11563475; doi:10.1371/journal.pbio.3002877)
Supplement: S3 Table — Table displays the ß estimates, standard errors (SE) as well as statistics from the mixed-effects model computed to assess the impact of age on instrumental learning biases. Here, the dependent variable was the probability of repeating the same response for a given cue P(repeat). Data and code to compute the statistics presented in this table is available at https://osf.io/mcx36/. (PDF) [file pbio.3002877.s004.pdf]

**Main effects**				
outcome valence	0.749	0.04	411.6	<.001 ***
action shown	-0.315	0.03	144.3	<.001 ***
outcome salience	0.027	0.02	2.2	0.1
age	0.219	0.09	6.2	0.01 *
**Interaction effects**				
outcome valence x action shown	0.031	0.02	2.5	0.1
outcome valence x outcome salience	0.173	0.04	24.6	<.001 ***
action shown x outcome salience	0.022	0.02	1.4	0.2
outcome valence x age	0.114	0.04	9.7	0.002 **
action shown x age	-0.054	0.03	4.5	0.04 *
outcome salience x age	0.007	0.02	0.2	0.7
outcome valence x action shown x outcome salience	0.063	0.03	6.1	0.01*
outcome valence x action shown x age	0.015	0.02	0.7	0.4
outcome valence x outcome salience x age	0.032	0.03	0.9	0.4
action shown x outcome salience x age	0.003	0.02	0.02	0.9
outcome valence x action shown x outcome salience x age	0.033	0.02	1.7	0.2
