# Supplementary material for: Decrease in decision noise from adolescence into adulthood mediates an increase in more sophisticated choice behaviors and performance gain
Source: PLoS Biol. 2024 Nov 14;22(11):e3002877. doi: 10.1371/journal.pbio.3002877 (PMC11563475; doi:10.1371/journal.pbio.3002877)
Supplement: S4 Table — Table providing an overview of the ß estimates, standard errors (SE) as well as statistics from the mixed-effects model computed assessing the impact of gender on the measured task effects. Here, the dependent variable was the probability of making a Go response P(Go). Data and code to compute the statistics presented in this table is available at https://osf.io/mcx36/. (PDF) [file pbio.3002877.s005.pdf]

| | ß estimates | SE | $\chi^2$ | p-value |
|---|---|---|---|---|
| **Main effects** | | | | |
| valence | 0.360 | 0.05 | 60.5 | <.001 *** |
| required action | 1.433 | 0.09 | 244.9 | <.001 *** |
| age | -0.019 | 0.07 | 0.09 | 0.8 |
| gender | 0.168 | 0.07 | 6.7 | 0.010** |
| **Interaction effects** | | | | |
| required action x valence | 0.161 | 0.04 | 13.7 | <.001 *** |
| valence x age | 0.103 | 0.05 | 5.05 | 0.03* |
| valence x gender | 0.086 | 0.05 | 3.5 | $0.06^+$ |
| required action x age | 0.210 | 0.09 | 5.3 | 0.02* |
| required action x gender | -0.123 | 0.10 | 1.8 | 0.2 |
| valence x required action x age | 0.082 | 0.04 | 3.6 | $0.06^+$ |
| valence x required action x gender | -0.003 | 0.04 | 0.005 | 0.9 |
