# Supplementary material for: Decrease in decision noise from adolescence into adulthood mediates an increase in more sophisticated choice behaviors and performance gain
Source: PLoS Biol. 2024 Nov 14;22(11):e3002877. doi: 10.1371/journal.pbio.3002877 (PMC11563475; doi:10.1371/journal.pbio.3002877)
Supplement: S5 Table — Table providing an overview of the ß estimates, standard errors (SE) as well as statistics from the mixed-effects model computed assessing the impact of gender on the instrumental learning bias. Here, the dependent variable was the probability of repeating the same response for a given cue P(repeat). Data and code to compute the statistics presented in this table is available at https://osf.io/mcx36/. (PDF) [file pbio.3002877.s006.pdf]

	ß estimates	SE	χ^2	p-value
**Main effects**				
outcome valence	0.749	0.04	411.6	<.001 ***
action shown	-0.317	0.03	150.8	<.001 ***
outcome salience	0.027	0.02	2.2	0.1
age	0.222	0.09	6.4	0.01 *
gender	-0.077	0.09	0.8	0.4
**Interaction effects**				
outcome valence x action shown	0.031	0.02	2.5	0.1
outcome valence x outcome salience	0.171	0.04	24.2	<.001 ***
action shown x outcome salience	0.022	0.02	1.4	0.24
outcome valence x age	0.114	0.04	9.7	0.002**
outcome valence x gender	-0.001	0.04	0.001	0.97
**action shown x age**	-0.057	0.03	5.1	0.02*
action shown x gender	0.049	0.03	3.6	0.06 $^{+}$
outcome salience x age	0.007	0.02	0.2	0.7
outcome salience x gender	0.002	0.02	0.008	0.9
outcome valence x action shown x outcome salience	0.061	0.03	6.0	0.01*
outcome valence x action shown x age	0.015	0.02	0.7	0.4
outcome valence x action shown x gender	-0.008	0.02	0.2	0.7
outcome valence x outcome salience x age	0.031	0.03	0.8	0.4
outcome valence x outcome salience x gender	0.038	0.03	1.2	0.3
action shown x outcome salience x age	0.0007	0.02	0.002	0.97
action shown x outcome salience x gender	0.018	0.02	0.9	0.3
outcome valence x action shown x outcome salience x age	0.030	0.02	1.5	0.2
outcome valence x action shown x outcome salience x gender	0.048	0.03	3.7	0.054 $^{+}$
