# Supplementary material for: Decrease in decision noise from adolescence into adulthood mediates an increase in more sophisticated choice behaviors and performance gain
Source: PLoS Biol. 2024 Nov 14;22(11):e3002877. doi: 10.1371/journal.pbio.3002877 (PMC11563475; doi:10.1371/journal.pbio.3002877)
Supplement: Attachment — Submitted filename: ResponseToReviewers_PlosBio_Scholz_Deserno.pdf [file pbio.3002877.s015.pdf]

Dear Dr. Schnell,

Thank you for giving us the opportunity to revise and resubmit our manuscript. We thoroughly considered the concerns raised by both reviewers with regard to the initial submission. We have worked extensively to address these comments, which has led to an improved manuscript. We give detailed responses to every point in the following and refer to the revised manuscript accordingly.

bold = original reviewer comments
normal = our reply
“quotation” = copied section from the manuscript
blue = changed manuscript text

Reviewer #1:
The manuscript "Biased or noisy? Motivational biases and decision noise across development" describes a study that aims to investigate whether decision noise is related to motivational choice biases and whether these parameters vary across age. Results suggested that Pavlovian (but not instrumental) choice biases and decision noise (referred to as feedback sensitivity) both correlate with age. Interestingly decision noise was found to mediate the effect of age on task performance. Finally, the study showed that decision noise as measured in the primary task correlated with decision noise parameters derived from other reinforcement learning tasks. By contrast, learning rates measured across the three learning tasks did not correlate. This study asks interesting questions, is well designed and has broadly been conducted thoroughly. The findings are important in the adolescent literature but also in the wider learning and decision-making field which is grappling with the content and context specific nature of some of its computational parameters. I am happy to support the study to publication, however I have a small number points for either clarification or minor concerns.
We thank the reviewer for the overall positive evaluation of our work.

Analysis/Results

1. **In the first task the authors show that two parameters of five from Model 7 correlate with age. Should the correlations between model parameters and age be Bonferroni corrected for multiple comparisons? Multiple comparisons have been corrected when analysing the RL task but not (as far as I can tell) the Go/NoGo task. Should alpha not be set to 0.01? And if so, does that mean that the authors should report the correlation between decision noise and age as significant but that it does not survive Bonferroni correction. This may have further implications for the strength of evidence the conclusions are based on.**
   We thank the reviewer for pointing this out and apologize for not having explained the rational sufficiently.

   The assessment of the correlations between age and the Pavlovian bias parameter, specifically more choice biases with increasing age, as well as age and the feedback noise parameters, here less noise with increasing age, replicates previous reports (Eckstein et al., 2022; Raab & Hartley, 2020; Waltmann et al., 2023; Xia et al., 2021). Thus, we considered this a confirmatory analysis of an a-priori hypothesis. Thus, correction for multiple comparison is not required in our opinion here. For completeness, we report additional correlations between age and other parameters, the gobias and the learning rate, which are all non-significant.

   In contrast, the same strength of a priori hypothesis did not apply to the remaining analysis, which we considered more exploratory and for which we have employed conservative multiple comparison correction throughout. We have now made this rational clearer in the main text as follows:

Main manuscript methods (p. 22, ll. 510 - 516)

As we were particularly interested in the developmental changes underlying decision noise and biases in choice behaviour (Eckstein et al., 2022; Raab & Hartley, 2020; Waltmann et al., 2023; Xia et al., 2021), we evaluated these associations for the respective computational parameters from the winning model with age using spearman correlation coefficients. Given our specific hypotheses regarding the developmental pattern of those three parameters (rho_{+FB}, rho_{-FB} and Pavlovian bias), we considered these confirmatory analyses for which we did not apply multiple comparison correction. For completeness, we also report associations for age with the remaining two parameters, namely the learning rate and the Gobias in the supplement.

2. **One key question posed by the authors is "Pavlovian biases or decision noise - what is driving performance". However, the analyses conducted here may not fully answer the question. The analyses examine the mediation effect of decision noise on age in explaining performance but do not, as far as I can tell, ask whether these effects are further mediated by the Pavlovian bias parameter/measure. Could the authors clarify whether this analysis does fully address this question and if not remedy this.**
After careful consideration of the feedback by both reviewers, we have concluded that the way in which we previously presented the results led astray from our core research questions. Our main focus is now clearly on the mediation effects of noise on distinct aspects of higher-level performance instead of comparing effects of those sub-components. We have now deleted all references made to this particular question and have extensively rewritten and restructured the entire manuscript, in particular the introduction, result and discussion sections. We therefore consider the question "Pavlovian biases or decision noise - what is driving performance", no longer a central component of the manuscript and thus abstained from further pursuing additional analysis. Instead, we elaborated more on the mediation analyses and added additional parameters from computational models from both RL tasks, the reversal learning task as well as the two-step task.

3. **Given differences in age of onset of puberty, which can have an effect on learning and decision making in adolescence, can I ask whether gender was accounted for or regressed out of the models?**
In the initial manuscript, our mixed-effect model analysis only accounted for the age effect, as this was our main research question. However, we agree with the reviewer that indeed gender could exert additional, relevant effects on learning and decision processes and thus, we computed those models and added the results very concisely to the main manuscript as well as in more detail to the supplement material.

Main manuscript methods (p. 20, ll. 456 - 458))
Given previous evidence of differences in age of onset of puberty (d'Acremont & Van der Linden, 2006), we also ran models including gender as additional control variable, thereby assessing potential effects of gender on choice behaviour and biases.

Main manuscript results (p. 5, ll. 135 -139))
[…] Meanwhile, the selective impact of instrumental learning biases on choices did not relate to age (age x action taken x outcome valence x outcome salience: ß = 0.033, SE = 0.03, $\chi^2(1) = 1.73$, p = 0.2) (Figure 1E) (see SI for more details, model statistics and control analyses regarding gender, which did not influence results).

Supplement (p. 5-6, ll. 101 - 119)

Control analysis: Gender effects

To verify potentially relevant effects of gender on the effects of interest, namely the impact of age on Pavlovian and instrumental learning biases, we assessed two additional models as follows:

Pavlovian Bias:

DVgo ~ valence*action*(age_z + gender) + (1+valence*action|ID)

Instrumental learning bias:

P(repeat) ~ action taken $_{t-1}$ * outcome valence $_{t-1}$ * outcome salience $_{t-1}$ *(age_z + gender) + (action taken $_{t-1}$ * outcome valence $_{t-1}$ * outcome salience $_{t-1}$ + 1|Subject)

In each case, the pattern and direction of the main and interaction effects did not change and none of the effects significantly interacted with gender (Table S4 and S5).

Table S4: Effects of gender on Pavlovian biases

	ß estimates	SE	χ^2	p-value
**Main effects**				
valence	0.358	0.05	7.7	<.001 ***
required action	1.424	0.09	15.5	<.001 ***
age	-0.021	0.07	-0.3	0.7
gender	0.167	0.07	2.5	0.01*
**Interaction effects**				
required action x valence	0.160	0.04	3.6	<.001 ***
valence x age	0.104	0.06	2.3	0.03*
valence x gender	0.087	0.09	1.9	0.06^{+}
required action x age	0.218	0.09	2.4	0.02*
required action x gender	-0.113	0.10	-1.2	0.2
valence x required action x age	0.083	0.04	1.9	0.06^{+}
valence x required action x gender	-0.002	0.04	-0.1	0.96

Table S5: Effects of gender on Instrumental learning biases

	ß estimates	SE	χ^2	p-value
**Main effects**				
outcome valence	0.3749	0.04	20.3	<.001 ***
action shown	-0.317	0.03	-12.3	<.001 ***
outcome salience	0.027	0.02	1.5	0.1
age	0.222	0.09	2.5	0.01 *
gender	-0.077	0.09	-0.9	0.4
**Interaction effects**				
outcome valence x action shown	0.031	0.02	1.6	0.1
outcome valence x outcome salience	0.171	0.04	4.9	<.001 ***
action shown x outcome salience	0.022	0.02	1.2	0.24
outcome valence x age	0.114	0.04	3.1	0.002**
outcome valence x gender	-0.001	0.04	-0.03	0.97
**action shown x age**	-0.057	0.03	-2.3	0.02*
action shown x gender	0.049	0.03	1.9	0.06 $^{+}$
outcome salience x age	0.007	0.02	0.4	0.7
outcome salience x gender	0.002	0.02	0.1	0.9
outcome valence x action shown x outcome salience	0.061	0.03	2.5	0.01*
outcome valence x action shown x age	0.015	0.02	0.8	0.4
outcome valence x action shown x gender	-0.008	0.02	-0.4	0.7
outcome valence x outcome salience x age	0.031	0.03	0.9	0.4
outcome valence x outcome salience x gender	0.038	0.03	0.04	0.3
action shown x outcome salience x age	0.0007	0.02	0.04	0.97
action shown x outcome salience x gender	0.018	0.02	0.98	0.3
outcome valence x action shown x outcome salience x age	0.030	0.02	1.2	0.2

outcome valence x action shown x outcome salience x gender	0.048	0.03	1.9	0.054⁺

4. **Does feedback sensitivity for positive and negative events correlate across individuals? I would be interested in further disentangling these two mechanisms and evidence that they are not correlated across participants could help determine whether they are mediated by the same or distinct processes**.
We thank the reviewer for this interesting suggestion. Indeed, both feedback sensitivity parameters correlate with each other [r_s (90) = -0.44, p < 0.001]. This is plausible because both capture noise (or stochasticity) in choice following positive and negative feedback, respectively. In our case only positive feedback sensitivity with age. While it is conceivable (and potentially also indicated by the moderate correlation between both parameters) that both parameters capture distinct processes to some degree, we think this will be work in its own right and would remain too speculative as part of the present work.

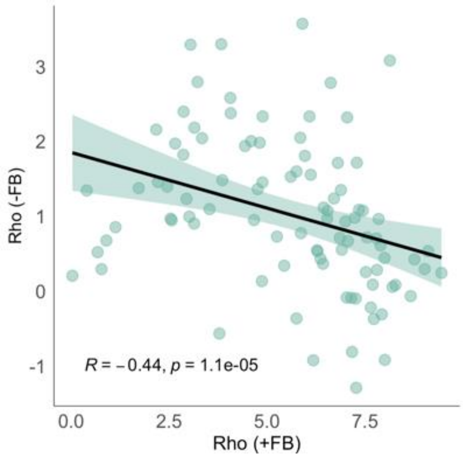

Introduction/Discussion

1. **My reading of the discussion on page 21 suggests that the authors initially suggest that the feedback sensitivity parameter is related to learning noise. However, later, on page 22, they note that the negative correlation between feedback control for negative outcomes and MB control could suggest that the decision noise in their study might be related to the directed component of exploration. Potentially this latter argument may be more in line with the current literature that suggest that directed exploration but not random exploration correlates with age (Sommerville et al 2017). Clarity on what the authors relate their decision noise parameter to in the context of exploration and the link to the Somerville conclusions would be appreciated.**
Due to some minor changes in the modelling analysis, namely we realized that there was a more appropriate way of initializing the choice values, the parameter estimates of the reported model changed slightly. Consequently, the previously reported correlation between sensitivity to negative feedback and model-based control is no longer significant. Thus, we refrain from further analyses or interpretations in this direction.

2. **I am unconvinced that the decrease in decision noise across adolescence chimes with the accounts of more limited availability of cognitive resources in adolescents. I suspect this may be context and content dependent. For example, there are some theories that suggest in some social, cognitive tasks adolescents use more computationally demanding strategies. For example, when mentalising, adolescents may have construct new schema on each occasion whereas adults are able to use previously stored schemas (Burnett et al. 2009). Similar short cuts and the use of decision heuristics may be taken by adults in other domains.**

We thank the reviewer for pointing this out. We fully agree with the reviewer's view that the claim we made about limited availability of cognitive resources being the most likely cause for the reported decrease in decision noise across adolescence, was limited. It fails to address important other explanations of our findings that are equally conceivable. We now rewrote the respective section to account for this and include a more in depths discussion of potential origins of our findings in the discussion instead of the introduction as follows (p. 14, ll. 299 - 316):

[…] One reason for these mediation effects could be a limited availability of cognitive resources in adolescents (Findling et al., 2019; Findling & Wyart, 2021; Juechems et al., 2021; Polanía et al., 2019) due to the ongoing development of brain areas related to cognitive control (Blakemore, 2012; Tamnes et al., 2013; Ziegler et al., 2019). Having fewer cognitive resources might make adolescents more prone to rely on computationally cheaper decision strategies, rendering them more susceptible to emotional, motivational and social influences (Gregorova et al., 2022; Reiter et al., 2017; Somerville & Casey, 2010). Still, other work has shown adolescents to employ more complex strategies relative to adults when mentalizing and processing social emotions (Burnett et al., 2009). The exertion of cognitive control as value-based choice, i.e., the willingness to allocate and exert control in certain situations (Cools, 2016, 2019; Kool et al., 2010; Lieder et al., 2018; Shenhav et al., 2013; Westbrook et al., 2013) might be another possible explanation. Here, cognitive control is a choice, governed by a cost-benefit tradeoff, where people choose to exert control whenever this will result in a large enough increase in expected reward. Individuals can thus learn to selectively exert control, when this returns additional reward, but refrain from it if costs outweigh the benefits (Chong et al., 2017; Manohar et al., 2015). More noisy choice behaviour, i.e. rather random-like exploration, may thus constitute a somewhat 'rational' choice by adolescents to not mobilize control to reduce effort expenditure while achieving higher levels of control, may seem too costly. Alternatively or in addition, Ma and colleagues (2022) suggested that elevated explorative (or noisier) behavior might serve the development of central social behavior in adolescence (Ma et al., 2022), such that choice uncertainty (comparable to decision noise) predicts contagion effects of peer's choices, which may be beneficial for social integration (Reiter et al., 2021).

3. **I spotted a number of typos in the manuscript. E.g. heard not heart (page 25), potentially missing the word "models" after "several hierarchically nested reinforcement learning" (page 27) and winnign (page 41). I would recommend a careful proofread.**
   We really appreciate the reviewer taking the time to go over the manuscript in detail. We apologize have now careful proofread the manuscript.

Reviewer #2:
In this paper, Scholz and colleagues examine the relations between developmental changes in decision noise (as indexed by positive and negative feedback sensitivity) and developmental changes in Pavlovian and instrumental learning biases from adolescence to adulthood. In the main, Pavlovian learning task, the researchers found that Pavlovian biases were lowest in adolescence but that positive feedback sensitivity increased with age. "Decision noise" related to performance but did not relate to Pavlovian or instrumental learning biases. The same group of participants also completed a reversal-learning task and the 'two-step' task, which measures model-based learning. Positive feedback sensitivity partially mediated the observed age-related increase in model-based control. Negative feedback sensitivity negatively predicted model-based control.
In general, while I believe this study has many methodological strengths, I found the framing, hypotheses, and takeaway somewhat muddled. In theory, addressing questions about the relation between decision noise and other types of learning biases

across development is very important, and I am glad the researchers aim to tackle this question. However, much of the logic in the introduction, results, and discussion was very difficult to follow / process. Part of this is due to a lack of specificity in language / terms used (which I detail below), and part of it seems to be due to a lack of conceptual clarity in the overall approach. It was not clear to me what question the experiments aimed to address or what the broader conclusions are. It seems like the main findings are that:

1. Pavlovian learning biases increase from adolescence into adulthood (which was reported in Raab & Hartley, 2020)

2. Decision noise is correlated across experiments (which was reported in Eckstein et. al, 2022) and decreases from adolescence into adulthood (which has been found in many prior studies).

It is possible that this study makes the additional novel contribution of simultaneously examining developmental changes in Pavlovian learning biases while also examining motivational biases on instrumental learning, but I had trouble understanding this aspect of the experiment and why this control is essential for the interpretation of previously reported developmental findings.

Below, I lay out my specific concerns in more details, organized mostly based on the order in which I encountered them.
We thank the reviewer for this feedback and for outlining potential ways of improving the current version of this manuscript. We agree with the reviewer and thus revised the manuscript comprehensively with regards to language specificity, structuring of the presented arguments and, maybe most importantly, with respect to the main focus of the result and their novelty. Critically, we have now emphasized clearly that the central and novel element of the manuscript is indeed on the mediation analyses: noise mediates the correlation between age and central readouts from each of the three tasks reported. Meanwhile, we considerably shortened other sections of the previous version of manuscript that were merely describing replications and transferred most of these descriptions to the supplement material with the intent of further sharpening our main line of arguments.

1. The term "motivational choice bias" is used extensively throughout the manuscript, beginning in the abstract. However, it was very unclear to me what this term was meant to refer to. A motivational choice bias could reflect: an action-specific bias (e.g., a 'go' bias), a valence-specific bias (e.g., weighting positive feedback more than negative feedback), or a valence x action interaction bias (e.g., a Pavlovian bias). The authors should clarify what they mean by this term in each case that they use it.
We agree with the reviewer that the term motivational choice bias is too imprecise. We now specifically separate a non-selective Pavlovian choice bias from a selective instrumental choice bias as follows:

Introduction: P. 3, ll. 78 - 83

[…] Another example is the influence of motivation on choices (Betts et al., 2020; Raab & Hartley, 2020). Individuals can be strongly biased to respond with behavioral activation in a rewarding context, while punishment tends to facilitate behavioral inhibition (Boureau & Dayan, 2011; Dayan et al., 2006; Guitart-Masip et al., 2014; Swart et al., 2017). This so-called Pavlovian choice bias has been found to be reduced in adolescence relative to childhood and adulthood (i.e. a weaker behavioral activation to rewards and lower behavioral inhibition to punishments), which was suggested to result from an elevated exploratory drive in adolescents (Raab & Hartley, 2020).

Results: P. 5, ll. 125 - 133

Specific age-dependent developmental changes. We employed a recent variant of a motivational Go/NoGo task to tease apart nonselective Pavlovian influence (i.e. behavioral activation to reward as compared to inhibition to punishment) from selective instrumental

influence (i.e. repetition of selective actions after reward and selective avoidance after punishment) (for an in-depth task description refer to the methods and SI). Using mixed-effects models, we replicated an age-dependent increase of Pavlovian biases from adolescence into adulthood (Raab & Hartley, 2020): age predicted more activation (Go responses) to reward cues and more inhibition (NoGo responses) to punishment cues (age x valence: ß = 0.107, SE = 0.05, $\chi^2(1) = 5.25$, p = 0.02) (Figure 1D). […].

We also provide an additional figure panel A in the supplemental Figure S2 for further clarification of the two different motivational choice biases we refer to in this study.

Supplement Material Figure S1, Panel A (p. 2)

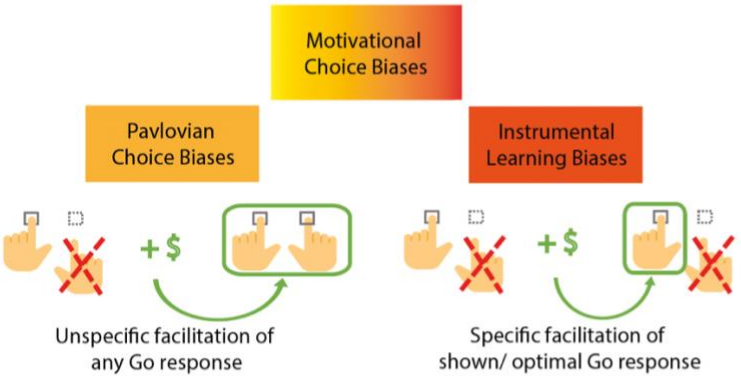

2. Similarly, I was confused by the use of the term "instrumental learning bias", which the authors seem to suggest is part of / or an example of a motivational choice bias. Is an instrumental learning bias increased weight on an instrumental (vs. Pavlovian) learning system, or is it a bias toward learning about left vs. right actions? (I think this is what the authors suggestion).
We agree with the reviewer that the terminology might have been too unclear in the previous version of the manuscript. As mentioned in the previous response, we now specifically define a non-selective Pavlovian choice bias from a selective instrumental choice bias and also added an additional panel A to Figure S2 in the supplement material for further clarification.

3. It was unclear to me why prior work suggesting heightened negative learning rates during adolescence would lead the researcher to hypothesize reductions in motivationally biased instrumental learning in adolescence, but this may in part stem from my confusion about their use of the phrase 'motivationally biased instrumental learning.' Why would learning more from negative outcomes lead to reduced selective response facilitation of the correct response for rewards?
We agree with the reviewer that this section was confusing. Given the extensive restructuring and resharpening of the focus of the manuscript, we dropped the section that you referred to and reformulated the hypotheses.

4. It would be helpful for the researchers to report more information about their age distribution. What proportion of the sample are 'adolescents'? A sample size of 93 seems relatively small given the wide age range, and the researchers may have been underpowered to detect developmental effects if the majority of their sample were adults (unless they had specific hypotheses about continued age-related change from age 18 - 45).
We now provide additional information about the proportion of "adolescent" participants, i.e. those aged 18 years or under. Specifically, 37 individuals of our sample were 18 years of age or younger (about 40%), while the remaining 56 individuals were above 18 years (60%). For a a better overview than a cut-off at 18 years, we also provided additional plots for age and gender distributions, which are now included in the supplemental Figure S1.

Main manuscript methods (p. 17, ll. 383 -385) and Figure in Supplement

[…] 40% (n = 37) of our final sample were adolescents (18 years of age or below). The age distribution was not uniform but right-skewed (see supplemental figure S1), as participants had been initially recruited as matched controls for a clinical sample in terms of age and gender.

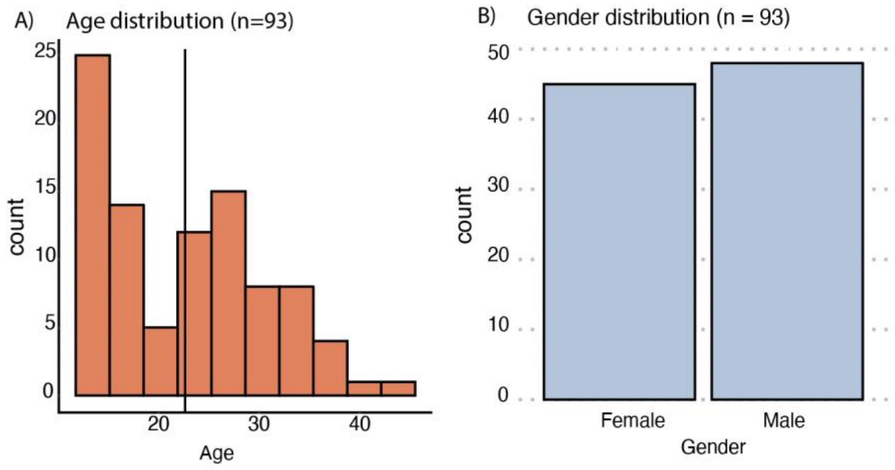

Figure S1: Distribution of Age and gender. **A) Age distribution** (age between 12-43 years) with the solid line indicating the mean age of 22.65 for this sample (n = 93). B) Gender distribution of sample. (Female = 45, Male = 48).

5. I think the mixed-effects results would be much easier to process if the researchers used 'accuracy' as a dependent variable instead of 'p(go)'. In general, it seems like the study aims to address how different types of learning biases and decision noise may contribute to age-related changes in learning performance. However, learning performance (as indexed by the proportion of 'correct' choices or the amount of reward earned) was never directly reported.

While we sympathize a lot with the idea of simplifying the manuscript, we do feel that the analysis using P(Go) as the dependent variable in the mixed effects models is the more consistent and intuitive measure here. This is based on frequent reports of it in the previous literature (Algermissen et al., 2022; Boer et al., 2018; Guitart-Masip et al., 2012; Scholz et al., 2022; Swart et al., 2017, 2018) and the fact that this style of analysis is indeed close to the computational model, which predicts Go vs. NoGo responses.

Nonetheless, we report learning performance as indexed by the proportion of correct choices overall. This could originally be found in the supplementary material (section *General task performance)*, which we have now shifted to the main results section (see p. 5, l. 135).

[…] This was accompanied by overall better performance with higher age ($r_s(91) = 0.24$, $p = 0.021$, overall task accuracy: mean [SD] = 66.24 [0.18], median [SD] = 69.69 [0.18]). […]

6. The results would be easier to parse if the researchers more explicitly described the results. For example, on lines 208 - 209, they write, "With increasing age, choices were more strongly impacted by motivational influences." However, it is difficult to understand how this maps on to the data. It would be more helpful to write something like, "With increasing age, participants demonstrated a stronger Pavlovian bias, such that they were more likely to 'go' following win versus loss cues."

We thank the reviewer for pointing this out and for the helpful suggestion of how to improve readability of the results. We now rewrote this sentence and also screened the manuscript for similar instances of imprecise descriptions of findings to address this problem. The following instances were corrected in the results section:

p. 5, ll. 130-133: […] Using mixed-effects models, we replicated an age-dependent increase of Pavlovian biases from adolescence into adulthood (Raab & Hartley, 2020): age predicted more activation (Go responses) to reward cues and more inhibition (NoGo responses) to punishment cues (age x valence: ß = 0.107, SE = 0.05, $\chi^2(1) = 5.25$, p = 0.02) (Figure 1D).

7. Raab and Hartley (2020) reported a quadratic effect of age on Pavlovian biases, not a nonquadratic age effect. Relatedly, it is unclear to me why so much space (Lines 209 - 220) is devoted to a non-significant age x action x valence interaction effect, when it seems as if the age x valence effect illustrates age-related change in Pavlovian biases.
We agree with the reviewer We have now dropped the discussion of this effect from the main manuscript and further information can only be found in the supplement on p. 3, ll. 59 - 66.

Table ST1: Mixed-effects model age dependent changes in Pavlovian biases

	ß estimates	SE	χ^2	p-value
**Main effects**				
valence	0.363	0.05	59.5	<.001 ***
required action	1.423	0.09	238.8	<.001 ***
age	-0.01	0.07	0.03	0.9
**Interaction effects**				
required action x valence	0.161	0.04	13.6	<.001 ***
valence x age	0.107	0.05	5.3	0.02*
required action x age	0.210	0.09	5.0	0.03*
valence x required action x age	0.082	0.04	3.6	0.06+

As can be derived from the model statistics in table ST1, we see a trend for the three-way interaction between action x valence x age (Figure S3A). […]

8. I was confused by the 'assessment of instrumental learning bias' section in part because 'instrumental learning bias' was never clearly defined. In this task, I think a 'perfect' instrumental learner should learn equally from all outcomes. Is the 'bias' the fact that instrumental learning may be influenced by the salience of the reward outcome? If so, how does the inclusion of two different 'go' actions enable this to be measured? I understand that this logic is likely spelled out in prior papers that have used this task, but I believe the researchers need to repeat it here so that developmentalists from wide-ranging backgrounds can understand the analyses.
While we described the term instrumental learning bias in the method section in the context of the mixed-effects model analysis, we admit, that its definition in the introduction was not clear enough especially for readers not familiar with previous work using this task version. However, given the shift in focus and extensive rewriting of the entire introduction, we now primarily added a more extensive description of instrumental learning biases to the method section and to supplement including an illustrative figure.

Main manuscript method section (p.18, ll. 407 -427)

[…] The impact of choice biases is operationalized by how well participants learn to show or omit a response when facing a reward or punishment cue requiring a Go or NoGo choice as optimal response. Consecutive choices represent learning of optimal choices and are guided by probabilistic feedback (rewards / neutral feedback for reward cues; punishment / neutral feedback for punishment cues). Importantly, bias-congruent responding (Go response for a Go-to-Win cue; NoGo for Avoid Punishment cue) should be facilitated, i.e. participants show better performance on these trials, while bias-incongruent performance should be impeded.

To tease apart differences in Pavlovian choice from instrumental learning biases, this task version had two Go responses, Go Left and Right (Algermissen et al., 2021; Swart et al., 2017,

2018). This manipulation discerns whether, for instance, a previously rewarded optimal Go responses (e.g. Go Left) will be specifically reinforced and repeated or omitted more frequently in subsequent trials with the same cue (Algermissen et al., 2021; Swart et al., 2017). This specificity for the optimal (Go) response reflects an instrumental learning bias, which will increase throughout the task while participants learn the optimal cue response, while the impact of Pavlovian choice bias recedes across the task (Swart et al., 2017). Overall, instrumental learning biases are more selective when compared with Pavlovian biases.

Unlike the instrumental learning bias, the Pavlovian bias does not distinguish between the type of Go response, here Go Left or Go Right, in its facilitation effect, so any Go response followed by a reward will increase the likelihood that a Go response will be selected on the next trial this cue is presented. Also, Pavlovian biases usually do so as early as the first trial (Swart et al., 2017) . They are also characterized by a non-specific tendency to show more Go responses for Win vs. Avoid Punishment cues. […]

9. A more minor point: The authors should avoid using the phrase 'action shown' when they mean 'action taken' as 'shown' implies the action was cued.'

We thank the reviewer and changed this across the manuscript.

10. In general, I thought the modelling was well done and I appreciate the model validation results the authors include in the supplement. However, I have two minor modelling points: First, rather than including two cue- or feedback-based reward sensitivity parameters, did the authors consider fitting a model with a single inverse temperature parameter? Such a model could perhaps similarly capture age related change in decision noise with fewer parameters.

We agree with the reviewer that first fitting more parsimonious models before moving on to more complex models is an essential computational modelling practice. Indeed, we had already included such a model with a single noise feedback sensitivity parameter (across both feedback types) in our model space, namely model M5, which is mathematically equivalent to the commonly used inverse temperature (see Katahira and colleagues (2015)). Both parameters, the feedback sensitivity as well as the inverse temperature parameter, capture the sensitivity of the choice probabilities to differences in value. Formally, the inverse temperature does so by determining the steepness of the softmax function. The feedback sensitivity parameter achieves this by computing the maximum difference between expected values, thus imposing a lower bound on choice stochasticity (Katahira, 2015; Waltmann et al., 2022). Thus, they are equivalent in their impact on choice probabilities.

Independent work from our group on the reliability of computational parameters from a reversal learning task (comparable to the one reported in this manuscript), showed that the models with reinforcement sensitivities not only outperformed models including inverse temperatures with regard to model comparison but also yielded higher reliability of computational parameters (Waltmann et al., 2022).

11. Second, the authors examine age-related change in parameter estimates using estimates from the hierarchically fitted model. This is statistically invalid, as the hierarchical fitting induces non-independence among the observations (which is required for linear regression to be valid). Rather than using the fitted parameters derived from the hierarchical fits, the researchers should use the fitted parameters from the first-level model fits in which parameter estimates are not influenced by those of other participants.

We admit that we disagree here. We will subsequently outline our line of reasoning:

First, these hierarchical models are set up to improve parameter estimation through regularization. Indeed, the superiority of such regularized parameters by means of identifiability, recovery and reliability has been shown in multiple studies using simulations as well as empirical data mainly through reduction of error variance (Katahira, 2015; Piray et al.,

2019; van Geen & Gerraty, 2021; Waltmann et al., 2022). In the same vein, the reviewer's argument would imply that using individual random slopes (as commonly extracted from mixed effects models, citation) would be invalid (Gillan et al., 2016; M Rouault, 2018; TXF Seow, 2020). Using non regularized parameter estimates (e.g. from the first step of estimation), potentially implicated by the reviewer, would decrease stability of parameter estimation considerably and forego the advantages of hierarchical modelling. This is also outlined in the methods paper of the authors of the cbm toolbox, which we use here (Piray et al., 2019).

Second, it is indeed the case that these parameters are no longer fully independent because they are regularized by an empirical prior distribution. However, if this prior does not contain any information about variables of interest for hypothesis testing (i.e. age in our case or group membership in clinical studies), these estimates remain unbiased for the hypotheses tested as in our case. Thus, in our case, the parameter estimates will be biased towards the overall grand mean (as reflected in the empirical priors), which can "pull" estimates closer together. If anything, this makes hypothesis testing on these parameters more conservative.

Third, there might be a misunderstanding that the inclusion of other models in the model space and during estimation (as implemented in the cbm toolbox) would modify the parameters and make them dependent on each other across different type of models, see Piray et al. for details. After estimating all models in one go for model selection, we estimated a single hierarchical model for parameter extraction of the winning model.

12. The section "Pavlovian biases or decision noise — what is driving performance?" was confusing to me because the age-related change in these parameter estimates make predictions in opposite directions. In the learning task, both greater decision noise and higher Pavlovian biases should potentially interfere with performance (at least on 50% of trials). Therefore, adolescents' reduced Pavlovian bias should lead to better performance and higher decision noise should lead to worse performance. In addition, it didn't seem like the effects of Pavlovian bias on performance was actually tested?
It was also unclear to me why the researchers expected the relation between age and Pavlovian bias to be mediated by feedback sensitivity, especially because again, they seem to make predictions in the opposite direction? Maybe I am missing something but I was confused by the logic here, as well as by the central question these analyses aimed to address. Similarly, in the discussion (lines 140 - 142), the researchers write, "As previous findings of a reduction in Pavlovian biases in adolescence might in part also have originated from worse overall task performance due to higher levels of decision noise during this developmental period …" but previous findings of a reduction in Pavlovian biases in adolescence led to better performance in adolescents relative to adults (Raab & Hartley, 2020).
We agree with the reviewer that the way we had structured our initial manuscript was misleading regarding the main research questions. Our main goal was to focus on the mediating effects of noise as well as its cross-task generalizability. By extensively restructuring and rewriting the entire manuscript, including most of the introduction, the result section as well as parts of the discussion, we aimed to remove the confusion. Further, we dropped the entire section that posed the question of "Pavlovian biases or decision noise — what is driving performance?", as we realized that this section led astray from the actual focus of this paper.

13. Did the researchers only look at correlations between positive feedback sensitivity in the Pavlovian learning task and feedback sensitivity in the reversal learning task? What about negative feedback sensitivity in the Pavlovian learning task?
In fact, we had initially assessed correlation coefficients between both feedback sensitivities for positive and negative outcomes from the Pavlovian bias task (2) and all feedback sensitivity parameters (4) from the reversal learning task and corrected them for multiple comparison by dividing the alpha level of 0.05 by 8, as 8 correlations were computed. However, only

correlations with the feedback sensitivity parameter for positive outcomes from the Pavlovian bias task were significant and thus reported as such.

Because this task version of reversal had an additional factor valence (gaining rewards vs. avoiding punishments), which is not of interest for the present analyses, we have now further simplified the correlational analysis: we averaged the 4 noise parameters from the reversal learning task across valence. On the one hand, this makes the parameters from the reversal task in this analysis more similar to the one we extracted from the model for the motivational Go/NoGo task. On the other hand, this reduces the number of computed correlations, from 8 to 4 in total (rho $_{+\,FB\,(Reversal)}$ x rho $_{+\,FB\,(GNG)}$; rho $_{-\,FB\,(Reversal)}$ x rho $_{Win\,+\,FB\,(GNG)}$; rho $_{-\,FB\,(Reversal)}$ x rho $_{-\,FB\,(GNG)}$; rho $_{+\,FB\,(Reversal)}$ x rho $_{Win\,-\,FB\,(GNG)}$). Multiple comparison correction was adjusted accordingly. The manuscript was modified as follows:

Main manuscript method section (p. 23, ll. 522 - 539)

Cross task generalizability of unspecific decision noise

Given our primary interest in the cross-task generalizability of decision noise, we subsequently assessed whether feedback sensitivity parameters from the motivational Go/NoGo task would be associated with related parameters from two other RL tasks detailed results for which have been published elsewhere (Scholz et al., 2023; Waltmann et al., 2023). Apart from the motivational Go/NoGo task, we had access to data from a probabilistic reinforcement learning task capturing cognitive flexibility and a sequential probabilistic decision-making task assessing model-based control (see SI for details on task design).

We first examined the association between computationally modelled noise parameters across the Go/NoGo task (ρ_{+FB} and ρ_{-FB}) and the probabilistic reversal learning task using spearman correlations. For the reversal task, noise parameters were extracted from the winning model, that had contained 4 noise parameters accounting for both outcome and cue valence, namely $\rho_{Win\,+FB}$, $\rho_{Win\,-FB}$, $\rho_{Loss\,+FB}$ and $\rho_{Loss\,-FB}$. Those estimates were further simplified by averaging parameters on the dimension of cue valence, thereby creating two parameter estimates, namely, $\rho_{Reversal\,+FB}$, $\rho_{Reversal\,-FB}$. In total, this meant that 4 correlations were computed across both tasks. Second, we also assessed cross-task associations for noise parameters from the Go/NoGo task (ρ_{+FB} and ρ_{-FB}) and the 2-step task, for which noise parameters ß1 and ß2 were extracted from a well-established hybrid model (see Daw et al. 2011 for an extensive model description).

Main manuscript results (p. 8-9, ll. 188 - 218)

Noise generalizes across RL tasks. Next, we examined the cross-task generalizability vs. context-specificity of noise parameters across several RL tasks: the Go/NoGo task, a probabilistic reversal learning task (Waltmann et al., 2023) and two-step task (Scholz et al., 2023). For the reversal task, the winning model, as described in Waltmann et al. 2023, comprised four feedback sensitivity parameters accounting for effects of motivational context (win reward vs avoid punishment) and feedback valence (positive /+FB vs negative/ -FB). For simplicity, we averaged parameter estimates across motivational context, resulting in two estimates based on feedback valence. While $\rho_{Go/NoGo\,+FB}$ from the Go/NoGo task was positively associated with the reversal feedback sensitivity parameter for positive outcomes $\rho_{Reversal\,+FB}$ [$r_s(87) = 0.47$, $p < 0.001$], the reversal feedback sensitivity parameter for negative outcomes correlated negatively with it [$\rho_{Reversal\,-FB}$: $r_s(87) = -0.3$, $p = 0.004$) (Figure 3A). Both survived multiple comparison correction with p-value < .0125. No correlation between reversal task noise and feedback sensitivity for negative outcomes ($\rho_{Go/NoGo\,-FB}$) reached significance ($p > 0.1$).

For the two-step task, both noise parameters, ß1 and ß2, were significantly correlated with Go/NoGo decision noise for positive outcomes (Figure 3B). More decision noise in the Go/NoGo task was associated with more decision noise from the two-step task (ß1: $r_s(90) = 0.43$, p-value < .001; ß2: $r_s(90) = 0.52$, p-value < .001). The association between 2nd stage

decision noise, ß2, and Go/NoGo decision noise for negative outcomes was weaker and barely survived multiple comparison correction ($r_s(90) = -0.26$, p-value = .012). Cross-task correlation for learning rates from each task proved non-significant ($\varepsilon_{\text{Go/NoGo}}$ X $\varepsilon_{\text{Reversal}}$: $r_s(87) = -0.027$, p = 0.8; $\varepsilon_{\text{Go/NoGo}}$ X $\varepsilon_{\text{TST alpha1}}$: $r_s(90) = -0.008$, p =0.94, ; $\varepsilon_{\text{Go/NoGo}}$ X $\varepsilon_{\text{TST alpha2}}$: $r_s(90) = -0.04$, p =0.74), highlighting the task- or context-specificity of those parameters.

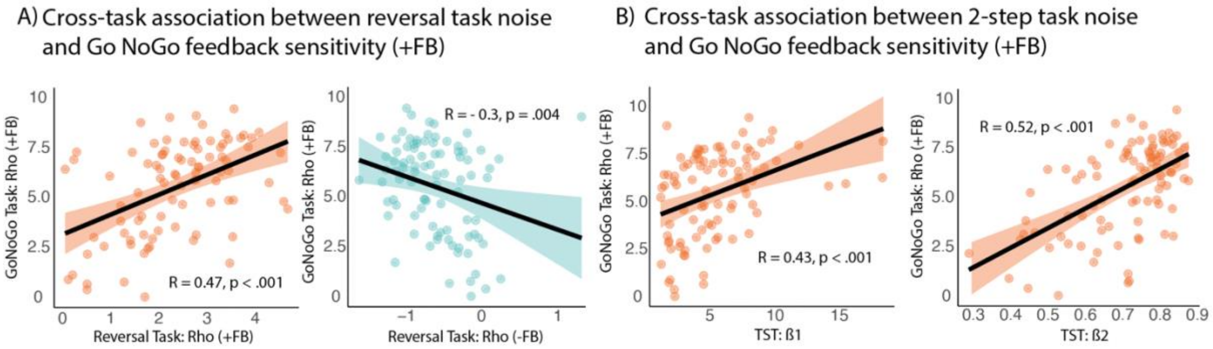

Figure 3 A) Depiction of the association between feedback sensitivity for positive outcomes from the Go/NoGo Task and noise parameters from the reversal task. As expected, all noise parameters which captured noise related to positive outcomes were positively correlated, while negative correlations could be observed when correlating feedback sensitivity for positive outcomes with feedback sensitivity parameters from the reversal task capturing feedback sensitivity for negative outcome B) Significant cross-task correlation between both computationally derived noise parameters ß1 and ß2 from the two-step task with the feedback sensitivity parameter for positive outcomes from the Go/NoGo Task. Results were considered significant with p < 0.05/4 (0.0125) respectively thereby correcting for multiple testing.

Results of additional correlational analyses of unspecific noise from the motivational Go/NoGo task with general and more specific task performance indices across all three tasks are reported in the SI. In short, less decision noise for positive outcomes in the Go/NoGo task was associated with increased overall accuracy on the Go/NoGo and reversal task (pre-post reversal accuracy) as well as more goal-directed behavior and less switching after negative outcomes in the reversal task. Meanwhile, less decision noise for negative outcomes in the Go/NoGo task was associated with decreased task performance in the GoNoGo task and in the reversal task.

14. I struggled to understand why the researchers included the model-based learning task results (which have already been reported in a published manuscript), when their primary focus seemed to be on the relation between decision noise and 'motivational choice biases.' It felt as if these results detracted from the central 'story' focused on Pavlovian and instrumental learning, and I was not sure what they aimed to illustrate by including them.

We think this impression was a result of the previous structure and focus of the manuscript. We had intended to focus on the specific mediation effects of noise on sophisticated choice processes while also testing noise generalizability across tasks. To clarify this focus, we have extensively restructured and rewritten the manuscript.

First, we added additional analyses, namely a cross-task generalizability analysis between noise parameters for the motivational Go/NoGo task with the 2-step task noise parameters (ß1 and ß2), extracted from a well-established hybrid model (Daw et al., 2011). This model was not part of our previous publication and represents a new analysis (Scholz et al. 2023).

Second, we also added mediation analyses, assessing the mediating effects of positive outcome noise on specific choice behaviour, such as reversal task performance and switching behavior after negative outcomes, both representing certain components of cognitive flexibility.

Third, we computed a mediation analysis for the computational parameter omega reflecting the balance between model-based and model-free behavior. Again, we found noise to be a significant mediator of the relationship between age and omega.

Main manuscript methods (p.p. 24-25, ll. 557 - 583)

Decision noise as a mediator for higher-level cognitive processes?

To address our second aim for this paper, we also computed several mediation analyses. All included feedback sensitivity as mediator variable and assessed its significance for age related changes on specific cognitive functions or decision processes, namely MB control, decision biases and markers of cognitive flexibility. The mediation package in R was used for all mediation analysis. Results are reported based on non-parametric bootstrap confidence intervals based on the percentile method (simulations n = 10.000).

The impact of decision noise on Go/NoGo task performance and Pavlovian biases

To determine whether previous findings of decreased Pavlovian biases reported during adolescence (Betts et al., 2020; Raab & Hartley, 2020) represented a partial masking of noise, we also assessed whether either of the two noise parameters extracted from our winning computational model (ρ_{+FB} and ρ_{-FB}) exerted a mediating effect on the relationship between age and overall task performance (P(correct), and on a previously employed score computed for the overall impact of Pavlovian biases on choice behaviour [P_{corr}(Go2Win) - P_{corr}(Go2Avoid) + (P_{corr}(NoGo2Win) - P_{corr}(NoGo2Avoid)] (Swart et al., 2017) as well as the computational parameter capturing Pavlovian biases.

The role of decision noise for the association between age and cognitive flexibility

Based on Waltmann et al. (2023), the difference between pre and post reversal accuracy as well as the degree of switching behaviour after negative feedback showed significant correlations with age. Hence, we examined whether those results were further impacted by the noise parameters for positive and negative outcomes from the motivational Go/NoGo task.

The impact of decision noise on the association between age and MB control

Finally, given previous reports linking age and MB control (Scholz et al., 2023), we also assessed whether this association might be (partially) mediated by the extracted noise parameters from our winning model M7, namely ρ_{+FB} and ρ_{-FB}. MB control was operationalized in two different ways, namely, the computationally derived parameter omega extracted from a well-established hybrid model (Daw et al., 2011) as well as effect estimates extracted from a mixed-effects model characterizing MB control (details reported in Scholz et al. 2023).

Main manuscript results (p. 9 -12, ll. 219 - 264)

Noise mediates developmental changes in decision processes. A key interest of our study was to examine whether decision noise mediates specific developmental changes in decision processes, critically, across tasks. We specified mediation effects of noise from the motivational GoNoGo task (feedback sensitivity for positive feedback) on the association between age and 1) general task performance and non-selective Pavlovian Bias on the Go/NoGo task, 2) performance and loose shift behaviour on the reversal task and 3) model-based behaviour on the 2-step task. This was done specifically for feedback sensitivity for positive feedback, given that feedback sensitivity for negative feedback showed no association with age.

Within-task mediation: motivational Go/NoGo task. We set up a mediation model with the age-dependent noise parameter (sensitivity to positive feedback) from the computational model of the motivational GoNoGo task as mediator of general performance in the task and Pavlovian bias. Assessing mediation effects of positive feedback sensitivity (noise) on the association between age and overall performance, we found a significant partial mediation effect for ρ_{+FB} (p= .02), accounting for 83,6 % of the total effect of the relationship between age and overall performance (Indirect effect = 0.004, CI [0.0005 – 0.01], p = .03; direct effect = 0.0009, CI [-0.001 – 0.003], p = .5; Total effect = 0.005, CI [0.0008 – 0.01], p = .02). Next, we examined whether the association between age and the score computed for overall Pavlovian bias [P_{corr}(Go2Win) - P_{corr}(Go2Avoid) + (P_{corr}(NoGo2Win) - P_{corr}(NoGo2Avoid)] or the computationally derived parameter capturing the Pavlovian bias, was mediated by positive feedback sensitivity. This association between age and Pavlovian bias was not significantly

mediated by positive feedback sensitivity, neither for the task score (p-value = 0.3) nor for the computational bias parameter (p-value = 0.5).

Across-task mediation: reversal task. Two mediation models were computed to determine the mediating effect of feedback sensitivity for positive outcomes (noise) in the GoNoGo task on performance parameters derived from the reversal learning task, namely pre- minus post-reversal accuracy and switching following negative outcomes, both of which have previously been shown to correlate with age (Waltmann et al., 2023). Assessment of the mediation effect of noise on the association between age and pre- minus post-reversal accuracy provided evidence for a partial mediation with ρ_{+FB} accounting for up to 37.1 % of the total effect (p = .03) (Indirect effect = 0.0016, CI [0.0002 – 0.004], p = .02; direct effect = 0.003, CI [-0.0004 – 0.01], p = .08; Total effect = 0.005, CI [0.001 – 0.01], p = .01) (Figure 4C). In the second mediation model, ρ_{+FB} accounted for up to 26,03 % of the total effect between age and switching after negative feedback (p = .02) (Indirect effect = - 0.002, CI [- 0.003 – -0.0002], p = .02; direct effect = 0.005, CI [- 0.007 – 0.001], p = .005; Total effect = - 0.006, CI [- 0.009 – - 0.003], p < .001) (Figure 4A).

Cross-task *mediation: 2-step task.* Assessment of the mediation effect of feedback sensitivity for positive outcomes in the GoNoGo task on the association between age and model-based control provided evidence of a partial mediation with ρ_{+FB} accounting for up to 44,01 % of the total effect of the relationship of age and model-based control (p = .02) (Indirect effect = 0.002, CI [0.0004 – 0.004], p = .02; direct effect = 0.003, CI [-0.0003 – 0.01], p = .08; Total effect = 0.005, CI [0.002 – 0.01], p = .004) (Figure 4D). Rerunning the same mediation model using the computationally derived parameter omega (weight parameter capturing the balance between model-free and model-based control) indicated a similar, though somewhat weaker mediation effect (19.75 %, p-value = .017) (see Figure 4B).

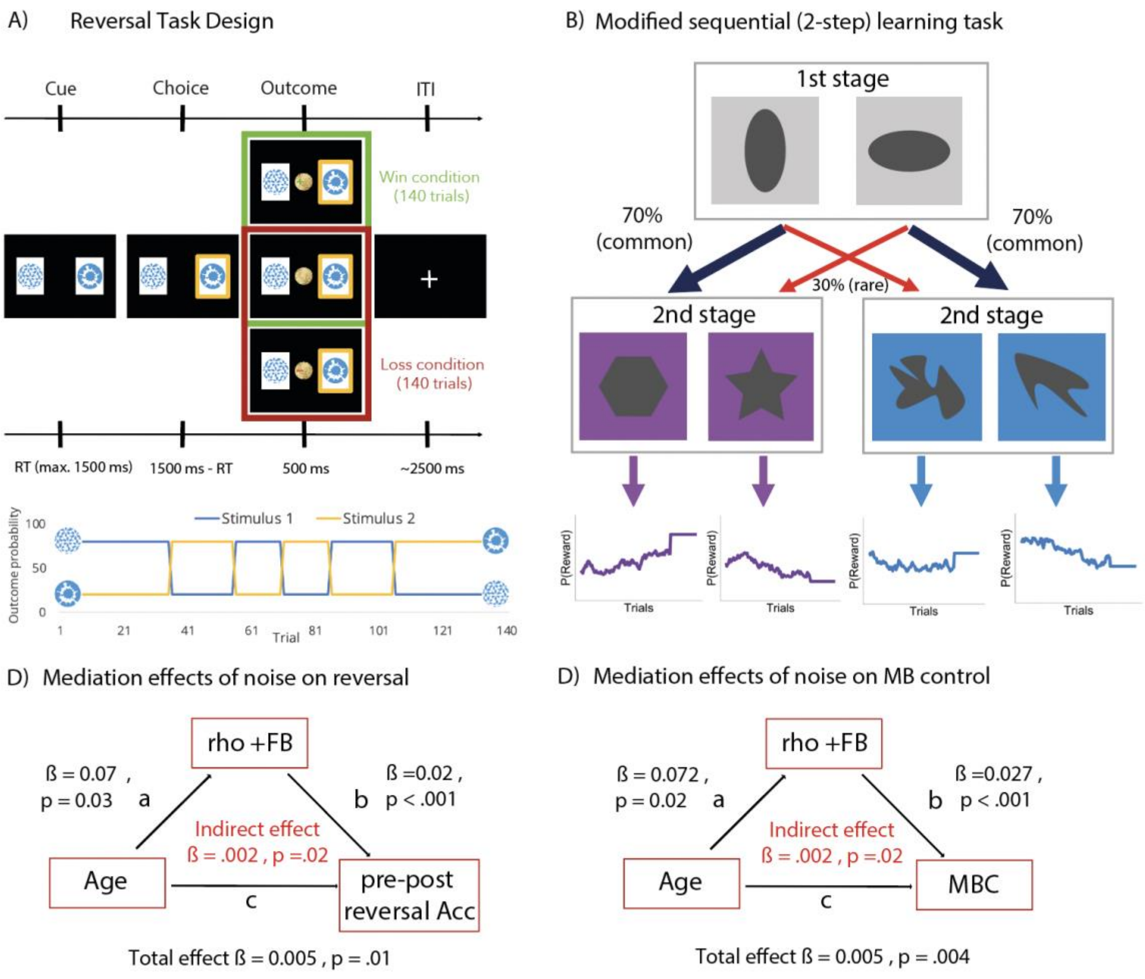

Figure 4 A) Probabilistic reversal task design. In this task participants had to adapt their behaviour according to the changing outcome probabilities across the reversal learning task. Image of task design modified from Waltmann et al. 2023. **B) Task design of sequential decision-making task.** In this „2-step" task, a choice on the first stage led to one of two possible second stages. Here, participants had to make a second choice, upon which participants received a reward or neutral outcome (rewards were replaced by punishments in the punishment context). The probability of receiving a reward/ punishment was determined by constantly changing probabilities, i.e. based on Gaussian random walks, while transition probabilities to transfer from stage 1 to stage 2 were fixed. They were either considered common transitions (70%) or rare (30%). Image modified from Scholz et al. 2023. C) Mediation analysis reversal task indicating a significant mediation effect of noise from positive outcomes on the association between age and pre minus post reversal accuracy. D) **Mediation analysis** 2-step task showing the significant mediation effect of feedback sensitivity for positive outcomes on the relationship between age and model-based control. P values below 0.025 were considered significant thereby correcting for multiple testing.

Main manuscript discussion (p. 13, ll. 266 - 273)

In this study, we relied on computational modelling across three distinct RL tasks to assess a novel mediating role of decision noise - known to be elevated in adolescents - on age-dependent increases in more sophisticated choice behaviors and performance gain. This mediating role referred to model-based control, switching after negative outcomes on the reversal task and performance gain in both the Go/NoGo and reversal task. A choice heuristic like the Pavlovian bias was not mediated by decision noise. In line with this mediating role of decision noise, we also confirm previous findings indicating decision noise as a rather stable characteristic across tasks and contexts.

Main manuscript discussion (p. 14, ll. 292 - 316)

Critically, the central novel finding of our study indicated a mediating effect of this seemingly 'unspecific' but stable noise on age-dependent increases in specific and more sophisticated choice signature across different task settings. Here, mediation analyses revealed that a substantial part of the variances between age and MB control, switching behaviour after negative outcomes as well as overall task performance on the reversal and Go/NoGo task were accounted for by age-related developmental differences in noise levels. Consequently, 'unspecific' noise mediates the development of highly specific functions or strategies.

One reason for these mediation effects could be a limited availability of cognitive resources in adolescents (Findling et al., 2019; Findling & Wyart, 2021; Juechems et al., 2021; Polanía et al., 2019) due to the ongoing development of brain areas related to cognitive control (Blakemore, 2012; Tamnes et al., 2013; Ziegler et al., 2019). Having fewer cognitive resources might make adolescents more prone to rely on computationally cheaper decision strategies, rendering them more susceptible to emotional, motivational and social influences (Gregorova et al., 2022; A. Reiter et al., 2017; Somerville & Casey, 2010). Still, other work has shown adolescents to employ more complex strategies relative to adults when mentalizing and processing social emotions (Burnett et al., 2009). The exertion of cognitive control as value-based choice, i.e., the willingness to allocate and exert control in certain situations (Cools, 2016, 2019; Kool et al., 2010; Lieder et al., 2018; Shenhav et al., 2013; Westbrook et al., 2013) might be another possible explanation. Here, cognitive control is a choice, governed by a cost-benefit tradeoff, where people choose to exert control whenever this will result in a large enough increase in expected reward. Individuals can thus learn to selectively exert control, when this returns additional reward, but refrain from it if costs outweigh the benefits (Chong et al., 2017; Manohar et al., 2015). More noisy choice behaviour, i.e. rather random-like exploration, may thus constitute a somewhat 'rational' choice by adolescents to not mobilize control to reduce effort expenditure while achieving higher levels of control, may seem too costly. Alternatively or in addition, Ma and colleagues (2022) suggested that elevated explorative (or noisier) behavior might serve the development of central social behavior in adolescence (Ma et al., 2022), such that choice uncertainty (comparable to decision noise) predicts contagion effects of peer's choices, which may be beneficial for social integration (A. M. F. Reiter et al., 2021).

Main manuscript discussion (p. 16, ll. 362 - 372)

In sum, using computational modelling and mediation analysis, we showed that decision noise had a significant mediating effect on age-dependent increases in higher-level cognitive processes such as model-based control, switching after negative outcomes in the reversal task and overall performance in the motivational Go NoGo and the reversal task. Cross-task analyses also emphasized decision noise as representing an inter-individually more stable parameter, maybe even a trait-like feature. Future work may unravel the neural basis as well as the developmental and clinical real-life relevance of decision noise for neurodevelopmental disorders such as ADHD to bridge the gap between observed symptom-level behavior and neurobiological mechanisms. Moreover, given that many of the cognitive processes we measure appear to be at least in part impacted by noise, future studies should attempt to quantify the degree of noise relative to the central cognitive processes under investigation.

References

Algermissen, J., Swart, J. C., Scheeringa, R., Cools, R., & den Ouden, H. E. M. (2021). Biased credit assignment in motivational learning biases arises through prefrontal influences on striatal learning. *bioRxiv Preprint*. https://www.biorxiv.org/content/10.1101/2021.10.03.462927v1

Algermissen, J., Swart, J. C., Scheeringa, R., Cools, R., & Den Ouden, H. E. M. (2022). Striatal BOLD and midfrontal theta power express motivation for action. *Cerebral Cortex*, *32*(14), 2924–2942. https://doi.org/10.1093/cercor/bhab391

Betts, M. J., Richter, A., de Boer, L., Tegelbeckers, J., Perosa, V., Baumann, V., Chowdhury, R., Dolan, R. J., Seidenbecher, C., Schott, B. H., Düzel, E., Guitart-Masip, M., & Krauel, K. (2020). Learning in anticipation of reward and punishment: Perspectives across the human lifespan. *Neurobiology of Aging*, *96*. https://doi.org/10.1016/j.neurobiolaging.2020.08.011

Blakemore, S. J. (2012). Imaging brain development: The adolescent brain. *NeuroImage*, *61*(2). https://doi.org/10.1016/j.neuroimage.2011.11.080

Boer, L. de, Axelsson, J., Chowdhury, R., Riklund, K., Dolan, R. J., Nyberg, L., Bäckman, L., & Guitart-Masip, M. (2018). Dorsal striatal dopamine D1 receptor availability predicts an instrumental bias in action learning. *Proceedings of the National Academy of Sciences*, 201816704. https://doi.org/10.1073/PNAS.1816704116

Boureau, Y. L., & Dayan, P. (2011). Opponency revisited: Competition and cooperation between dopamine and serotonin. *Neuropsychopharmacology*, *36*(1), 74–97. https://doi.org/10.1038/npp.2010.151

Burnett, S., Bird, G., Moll, J., Frith, C., & Blakemore, S.-J. (2009). Development during adolescence of the neural processing of social emotion. *Journal of Cognitive Neuroscience*, *21*(9), 1736–1750. https://doi.org/10.1162/jocn.2009.21121

Chong, T. T. J., Apps, M., Giehl, K., Sillence, A., Grima, L. L., & Husain, M. (2017). Neurocomputational mechanisms underlying subjective valuation of effort costs. *PLoS Biology*, *15*(2). https://doi.org/10.1371/journal.pbio.1002598

Cools, R. (2016). The costs and benefits of brain dopamine for cognitive control. *Wiley Interdisciplinary Reviews. Cognitive Science*, *7*(5), 317–329. https://doi.org/10.1002/wcs.1401

Cools, R. (2019). Chemistry of the Adaptive Mind: Lessons from Dopamine. *Neuron*, *104*(1), 113–131. https://doi.org/10.1016/j.neuron.2019.09.035

d'Acremont, M., & Van der Linden, M. (2006). Gender differences in two decision-making tasks in a community sample of adolescents. *International Journal of Behavioral Development*, *30*(4), 352–358. https://doi.org/10.1177/0165025406066740

Daw, N. D., Gershman, S. J., Seymour, B., Dayan, P., & Dolan, R. J. (2011). Model-based influences on humans' choices and striatal prediction errors. *Neuron*, *69*(6), 1204–1215. https://doi.org/10.1016/j.neuron.2011.02.027 PM - 21435563 M4 - Citavi

Dayan, P., Niv, Y., Seymour, B., & Daw, N. D. (2006). The misbehavior of value and the discipline of the will. *Neural Networks*, *19*(8). https://doi.org/10.1016/j.neunet.2006.03.002

Eckstein, M. K., Master, S. L., Dahl, R. E., Wilbrecht, L., & Collins, A. G. E. (2022). Reinforcement learning and Bayesian inference provide complementary models for the unique advantage of adolescents in stochastic reversal. *Developmental Cognitive Neuroscience*, *55*. https://doi.org/10.1016/j.dcn.2022.101106

Findling, C., Skvortsova, V., Dromnelle, R., Palminteri, S., & Wyart, V. (2019). Computational noise in reward-guided learning drives behavioral variability in volatile environments. *Nature Neuroscience*, *22*(12). https://doi.org/10.1038/s41593-019-0518-9

Findling, C., & Wyart, V. (2021). Computation noise in human learning and decision-making: Origin, impact, function. *Current Opinion in Behavioral Sciences*, *38*. https://doi.org/10.1016/j.cobeha.2021.02.018

Gillan, C. M., Kosinski, M., Whelan, R., Phelps, E. A., & Daw, N. D. (2016). Characterizing a psychiatric symptom dimension related to deficits in goal-directed control. *eLife*, *5*. https://doi.org/10.7554/eLife.11305 PM - 26928075 M4 - Citavi

Gregorova, K., Eldar, E., Deserno, L., & Reiter, A. M. F. (2022). *A cognitive-computational account of mood swings in adolescence*. https://doi.org/10.31234/osf.io/u62vr

Guitart-Masip, M., Duzel, E., Dolan, R., & Dayan, P. (2014). Action versus valence in decision making. *Trends in Cognitive Sciences*, *18*(4), 194–202. https://doi.org/10.1016/j.tics.2014.01.003 U6 - http://www.cell.com/article/S1364661314000205/fulltext M4 - Citavi

Guitart-Masip, M., Huys, Q. J. M., Fuentemilla, L., Dayan, P., Duzel, E., & Dolan, R. J. (2012). Go and no-go learning in reward and punishment: Interactions between affect and effect. *NeuroImage*, *62*(1), 154–166. https://doi.org/10.1016/j.neuroimage.2012.04.024 PM - 22548809 M4 - Citavi

Juechems, K., Balaguer, J., Spitzer, B., & Summerfield, C. (2021). Optimal utility and probability functions for agents with finite computational precision. *Proceedings of the National Academy of Sciences of the United States of America*, *118*(2). https://doi.org/10.1073/pnas.2002232118

Katahira, K. (2015). The relation between reinforcement learning parameters and the influence of reinforcement history on choice behavior. *Journal of Mathematical Psychology*, *66*, 59–69. https://doi.org/10.1016/j.jmp.2015.03.006

Kool, W., McGuire, J. T., Rosen, Z. B., & Botvinick, M. M. (2010). Decision Making and the Avoidance of Cognitive Demand. *Journal of Experimental Psychology: General*, *139*(4), 665–682. https://doi.org/10.1037/a0020198

Lieder, F., Shenhav, A., Musslick, S., & Griffiths, T. L. (2018). Rational metareasoning and the plasticity of cognitive control. *PLoS Computational Biology*, *14*(4), 1–27. https://doi.org/10.1371/journal.pcbi.1006043

M Rouault, T. S., CM Gillan, SM Fleming. (2018). Psychiatric symptom dimensions are associated with dissociable shifts in metacognition but not task performance. *Biol. Psychiatry*, *84*, 443–451.

Ma, I., Westhoff, B., & van Duijvenvoorde, A. C. K. (2022). Uncertainty about others' trustworthiness increases during adolescence and guides social information sampling. *Scientific Reports*, *12*(1), Article 1. https://doi.org/10.1038/s41598-022-09477-2

Manohar, S. G., Chong, T. T. J., Apps, M. A. J., Batla, A., Stamelou, M., Jarman, P. R., Bhatia, K. P., & Husain, M. (2015). Reward Pays the Cost of Noise Reduction in Motor and Cognitive Control. *Current Biology*, *25*(13), 1707–1716. https://doi.org/10.1016/j.cub.2015.05.038

Piray, P., Dezfouli, A., Heskes, T., Frank, M. J., & Daw, N. D. (2019). Hierarchical Bayesian inference for concurrent model fitting and comparison for group studies. *PLoS Computational Biology*, *15*(6), e1007043. https://doi.org/10.1371/journal.pcbi.1007043

Polanía, R., Woodford, M., & Ruff, C. C. (2019). Efficient coding of subjective value. *Nature Neuroscience*, *22*(1). https://doi.org/10.1038/s41593-018-0292-0

Raab, H. A., & Hartley, C. A. (2020). Adolescents exhibit reduced Pavlovian biases on instrumental learning. *Scientific Reports*, *10*(1). https://doi.org/10.1038/s41598-020-72628-w

Reiter, A., Heinz, A., & Deserno, L. (2017). Linking social context and addiction neuroscience: A computational psychiatry approach. *Nature Publishing Group*, *18*. https://doi.org/10.1038/nrn.2017.67

Reiter, A. M. F., Moutoussis, M., Vanes, L., Kievit, R., Bullmore, E. T., Goodyer, I. M., Fonagy, P., Jones, P. B., & Dolan, R. J. (2021). Preference uncertainty accounts for developmental effects on susceptibility to peer influence in adolescence. *Nature Communications*, *12*(1), Article 1. https://doi.org/10.1038/s41467-021-23671-2

Scholz, V., Hook, R. W., Kandroodi, M. R., Algermissen, J., Ioannidis, K., Christmas, D., Valle, S., Robbins, T. W., Grant, J. E., Chamberlain, S. R., & den Ouden, H. E. M. (2022). Cortical dopamine reduces the impact of motivational biases governing automated behaviour. *Neuropsychopharmacology*, *47*(8). https://doi.org/10.1038/s41386-022-01291-8

Scholz, V., Waltmann, M., Herzog, N., Reiter, A., Horstmann, A., & Deserno, L. (2023). Cortical grey matter mediates increases in model-based control and learning from positive feedback from adolescence to adulthood. *The Journal of Neuroscience*. https://doi.org/10.1523/jneurosci.1418-22.2023

Shenhav, A., Botvinick, M. M., & Cohen, J. D. (2013). The expected value of control: An integrative theory of anterior cingulate cortex function. *Neuron*, *79*(2), 217–240. https://doi.org/10.1016/j.neuron.2013.07.007

Somerville, L. H., & Casey, B. J. (2010). Developmental neurobiology of cognitive control and motivational systems. *Current Opinion in Neurobiology*, *20*(2). https://doi.org/10.1016/j.conb.2010.01.006

Swart, J. C., Frank, M. J., Määttä, J. I., Jensen, O., Cools, R., & den Ouden, H. E. M. (2018). Frontal network dynamics reflect neurocomputational mechanisms for reducing maladaptive

biases in motivated action. *PLoS Biology*, *16*(10), e2005979. https://doi.org/10.1371/journal.pbio.2005979

Swart, J. C., Froböse, M. I., Cook, J. L., Geurts, D. E. M., Frank, M. J., Cools, R., & den Ouden, H. E. M. (2017). Catecholaminergic challenge uncovers distinct Pavlovian and instrumental mechanisms of motivated (in)action. *eLife*, *6*, 1–36. https://doi.org/10.7554/eLife.22169

Tamnes, C. K., Walhovd, K. B., Grydeland, H., Holland, D., Østby, Y., Dale, A. M., & Fjell, A. M. (2013). Longitudinal working memory development is related to structural maturation of frontal and parietal cortices. *Journal of Cognitive Neuroscience*, *25*(10). https://doi.org/10.1162/jocn_a_00434

TXF Seow, C. G. (2020). Transdiagnostic phenotyping reveals a host of metacognitive deficits implicated in compulsivity. *Sci. Rep.*, *10*.

van Geen, C., & Gerraty, R. T. (2021). Hierarchical Bayesian models of reinforcement learning: Introduction and comparison to alternative methods. *Journal of Mathematical Psychology*, *105*, 102602. https://doi.org/10.1016/j.jmp.2021.102602

Waltmann, M., Herzog, N., Reiter, A. M. F., Villringer, A., Horstmann, A., & Deserno, L. (2023). Diminished reinforcement sensitivity in adolescence is associated with enhanced response switching and reduced coding of choice probability in the medial frontal pole. *Developmental Cognitive Neuroscience*, *60*. https://doi.org/10.1016/j.dcn.2023.101226

Waltmann, M., Schlagenhauf, F., & Deserno, L. (2022). Sufficient reliability of the behavioral and computational readouts of a probabilistic reversal learning task. *Behavior Research Methods*, *54*(6), 2993–3014. https://doi.org/10.3758/s13428-021-01739-7

Westbrook, A., Kester, D., & Braver, T. S. (2013). What Is the Subjective Cost of Cognitive Effort? Load, Trait, and Aging Effects Revealed by Economic Preference. *PLoS ONE*, *8*(7). https://doi.org/10.1371/journal.pone.0068210

Xia, L., Master, S. L., Eckstein, M. K., Baribault, B., Dahl, R. E., Wilbrecht, L., & Collins, A. G. E. (2021). Modeling changes in probabilistic reinforcement learning during adolescence. *PLoS Computational Biology*, *17*(7). https://doi.org/10.1371/journal.pcbi.1008524

Ziegler, G., Hauser, T. U., Moutoussis, M., Bullmore, E. T., Goodyer, I. M., Fonagy, P., Jones, P. B., Lindenberger, U., & Dolan, R. J. (2019). Compulsivity and impulsivity traits linked to attenuated developmental frontostriatal myelination trajectories. *Nature Neuroscience*, *22*(6). https://doi.org/10.1038/s41593-019-0394-3
